# Evaluation of BD Onclarity™ HPV Assay on Self-Collected Vaginal and First-Void Urine Samples as Compared to Clinician-Collected Cervical Samples: A Pilot Study

**DOI:** 10.3390/diagnostics12123075

**Published:** 2022-12-07

**Authors:** Marianna Martinelli, Chiara Giubbi, Illari Sechi, Fabio Bottari, Anna Daniela Iacobone, Rosario Musumeci, Federica Perdoni, Narcisa Muresu, Andrea Piana, Robert Fruscio, Fabio Landoni, Clementina Elvezia Cocuzza

**Affiliations:** 1 Department of Medicine and Surgery, University of Milano-Bicocca, 20900 Monza, Italy; 2 Department of Medical, Surgical and Experimental Sciences, University of Sassari, 07100 Sassari, Italy; 3 Division of Laboratory Medicine, European Institute of Oncology IRCCS, 20141 Milan, Italy; 4 Department of Biomedical Sciences, University of Sassari, 07100 Sassari, Italy; 5 Preventive Gynecology Unit, European Institute of Oncology IRCCS, 20141 Milan, Italy; 6 Department of Humanities and Social Sciences, University of Sassari, 07100 Sassari, Italy; 7 Clinic of Obstetrics and Gynecology, Department of Medicine and Surgery, San Gerardo Hospital, University of Milan Bicocca, 20900 Monza, Italy

**Keywords:** human papillomavirus, self-collection, vaginal self-sample, first-void urine, cervical cancer screening, diagnostic accuracy

## Abstract

The accuracy of available HPV molecular assays on self-samples needs to be evaluated as compared to clinician-collected samples. This pilot study aimed to investigate the BD Onclarity™ HPV assay on vaginal and first-void urine samples. Sixty-four women referred to colposcopy for cervical dysplasia performed a vaginal self-collection and provided a first-void urine sample, after informed consent. A cervical specimen was collected during the clinician examination. All samples were tested using BD Onclarity™ HPV assay on the BD Viper™ LT System. Overall positive agreement (OPA) between cervical and self-sample results was evaluated using Cohen’s kappa value (κ). Using a clinical cut-off of 38.3 Ct for HPV 16 and 34.2 Ct for other HR genotypes, compared to cervical sample, the self-collected vaginal sample OPA was 85.9%, and κ = 0.699. Without a clinical cut-off, the OPA was 95.3%, and the κ = 0.890. Data obtained comparing cervical and urine samples showed an OPA of 87.5% with a κ = 0.79 using a clinical cut-off, and an OPA of 90.6% with a κ = 0.776 without a clinical cut-off. Data showed a substantial agreement between both self-collected and clinician-collected samples. A specific clinical cut-off analysis should be considered based on type of sample analysed.

## 1. Introduction

Cervical cancer is one of the most important malignancies affecting women and caused about 342,000 deaths in 2020. It is well-known that this type of tumour is caused by persisting infection of high-risk human papillomavirus (hrHPV). Recently, the World Health Organization (WHO) developed a global strategy for cervical cancer elimination to be reached by 2030, and one of the points of this strategy is to reach the 70% of women screened using a high-performance test by the age of 35, and again by the age of 45 [1].

Eighty-five percent of cervical cancer deaths occur in developing countries, where it still represents the first leading cancer death cause. Self-sampling could be an additional strategy to reach unscreened and under-screened women, especially in middle- and low-income countries.

As of now, the global use of HPV self-sampling is still limited. Only 17 countries with identified screening programs recommend the use of self-sampling in primary screening or to reach non-responder women. However, the COVID-19 pandemic has accelerated worldwide self-sampling introduction, which is now considered an important strategy to increase screening coverage in the coming years [2].

The importance of self-sampling in improving adherence to cervical cancer screening has been well documented in the last few years, especially for women not participating in prevention programs due to different socio-cultural reasons [3,4]. The main barrier is related to the need for a physician or healthcare worker for cervical specimen collection [5]. The use of alternative and less invasive samples, such as self-collected vaginal and first-void urine samples, represents the best choice to overcome this issue.

Different devices are commercially available and seem to be suitable for this purpose. However, the accuracy of clinically validated PCR-based human papillomavirus detection kits on self-samples needs to be evaluated as compared to clinician-collected samples, as already reported in a recent meta-analysis by Arbyn et al. [6]. Previous studies have already shown that HPV testing conducted on vaginal self-samples has a similar sensitivity compared to testing on physician-collected cervical samples for the detection of cervical intraepithelial neoplasia grade 2 or higher (CIN2+) [6,7,8]. Nevertheless, the sample preparation and preanalytical processes used are highly different [9]. Different assays suggest in their manufacturer instructions to start from different specimen volumes for hrHPV detection, and this could influence the result obtained, especially for self-collected samples. Moreover, the results obtained from samples collected using dry vaginal swabs could be conditioned from the solution volume used for swab resuspension.

Urine seems to be a good, non-invasive, and more acceptable material for the detection of HPV and sexually transmitted infections [10,11,12]. Moreover, because first-void urine contains exfoliated cells from the cervix [13], it could be considered a specimen alternative to a clinician-collected cervical sample for the molecular detection of HPV. Furthermore, several studies have recently been published reporting consistent results from the use of urine samples for HPV detection [14,15,16,17,18]. Also for this kind of sample, the performance of urine-based HPV testing for CIN2+ detection is affected by the various HPV assays and non-standardized urine collection methods [14].

The objective of this pilot study was to evaluate accuracy of the BD Onclarity™ HPV assay on self-collected vaginal and first-void urine samples as compared to clinician-collected cervical samples.

The BD Onclarity™ assay has been internationally validated for liquid-phase cytology samples for use in primary HPV screening according to both the Mejer guidelines and the VALGENT genotyping protocol [19,20]; in this study we evaluated the performance of this test, already extensively studied in a screening setting, on self-collected samples in order to assess its usefulness to increase adherence to screening programs.

In particular, in this pilot study, we decided to use the same BD Onclarity™ protocol that is used for cervical specimens, without making changes, in order to discover whether the same protocol could also work for different samples compared to a liquid-phase cytology sample.

## 2. Materials and Methods

### 2.1. Study Design and Sample Collection

For this pilot study of diagnostic accuracy, a group of 64 women (mean age: 38.4 years) with a recent diagnosis of cervical dysplasia attending the Colposcopy Clinic of San Gerardo Hospital (Monza, Italy) were enrolled. The study protocol was approved by the Ethics Committee of the University of Milano-Bicocca, Monza, Italy (Protocol n. 0037320/2017 and 0086409/2018). All subjects provided written and informed consent to participate in the study. Patients were excluded in case of immunodeficiency, HIV infection, presumed or confirmed pregnancy, diagnosis of any malignancies, and/or chemotherapy in the previous 6 months.

All women were adequately informed about the study by Colposcopy Clinic staff and were asked to autonomously collect a vaginal self-sample using a FLOQSwab^®^ (5E046S, Copan, Brescia, Italy) and a first-void urine (FVU) sample using a Colli-pee^®^ 20 mL (Novosanis, Wijnegem, Belgium) before colposcopy examination. The information brochure of the study and the instructions on how to use the devices were given to all participants.

During the colposcopy examination, the gynaecologist collected one cervical sample using an L-shaped FLOQSwab^®^ (Copan). All physician-collected cervical and self-collected vaginal and urine samples were sent to the Clinical Microbiology Laboratory of the Department of Medicine and Surgery, University of Milano-Bicocca, Italy, for preanalytical processing within 24 h of collection.

### 2.2. Preanalytical Sample Processing

Cervical samples were collected using an L-shaped FLOQSwab^®^ (Copan, Brescia, Italy) and transported in a tube with 20 mL of PreservCyt^®^ Solution (HOLOGIC, Marlborough, MA, USA). All samples were well shaken using vortex for 30 seconds, and 1.5 mL aliquots were made.

Vaginal self-samples were obtained using a FLOQSwab^®^ (Copan, Brescia, Italy) and transported dry to the laboratory. Each specimen was suspended in 5.5 mL of PreservCyt^®^ Solution (HOLOGIC, Marlborough, MA, USA), and 5 aliquots were made.

The Colli-Pee^®^ 20 mL device allowed for capturing a first-void urine volume of 13 mL (+/− 2 mL), in a collection device containing 7 mL of preservative urine conservation medium (UCM), leading to a final volume of 20 mL (+/− 2 mL) [15]. Urine samples were aliquoted after arrival at the laboratory. One aliquot of each type of sample was sent to the Division of Laboratory Medicine of the European Institute of Oncology, Milan, Italy, for HPV testing.

### 2.3. BD Onclarity™ HPV Assay

The BD Onclarity™ HPV assay (BD, USA), which detects 14 high-risk (HR) genotypes, provides the capability of extended genotyping through individual detection of HPV 31, 51, and 52 (in addition to 16, 18, and 45) and pooled detection of 33/58, 35/39/68, and 56/59/66. An endogenous human beta-globin sequence is detected as a sample validity control, sample extraction, and amplification efficiency [20,21,22].

All samples were tested following manufacturer instructions: BD Onclarity™ uses 0.5 mL of sample (cervical, vaginal, or urine sample) which is added to a suitable solution produced by BD (the LBC tube) to reach a final volume of 2.2 mL, of which 0.8 mL of sample is automatically taken by the instrument to perform nucleic acid extraction using the extraction chemistry developed by BD (BD FOX ™). The extracted DNA is then eluted to a final volume of 400 microliters, and 50 microliters is automatically pipetted into each of the three wells containing the dried master mix in order to perform real-time PCR.

The clinical cut-off is set to be related to CIN2+ disease, and an algorithm verifies the adequacy of the sample using the amplification of the human beta-globin gene. In particular, the software interprets the amplification curves on the basis of the following threshold cycles (Ct): 38.3 Ct for HPV16, 34.2 Ct for other HR-HPVs, and 34.2 Ct for beta-globin. Samples which were judged invalid were re-tested a second time (9 cervical samples, 2 vaginal swabs, and 4 urines).

### 2.4. Statistical Analysis

The overall percentage agreement (OPA) between cervical and self-sample results was evaluated using Cohen’s kappa value (κ) using the GraphPad QuickCalcs software (updated in 2014, available at http://graphpad.com/quickcalcs accessed on 30 October 2022). Agreement was interpreted as slight (κ < 0.200), fair (0.200 < κ < 0.401), moderate (0.400 < κ < 0.601), good (0.600 < κ < 0.801), very good (0.800 < κ < 1.000), or perfect (κ = 1.000). Sample results were evaluated using two different cut-offs: (i) clinical cut-off of 38.3 Ct (cycle threshold) for HPV 16 and 34.2 Ct for other HR genotypes, as indicated in the package insert and (ii) without the clinical cut-off (accepting positivity up to 40 Ct).

## 3. Results

### 3.1. Study Population

The 64 women enrolled in this study were referred to colposcopy because of a recent diagnosis of cervical dysplasia confirmed by Pap smear examination. Clinical data regarding Pap test, colposcopy, and biopsy results are reported in Table 1. Twenty-three out of the sixty-four women showed abnormal colposcopy findings, and cervical guided biopsies were taken to histologically define the grade of the lesion; 18/23 (78.3%) biopsies showed the presence of a CIN2+ lesion.

### 3.2. HPV Positivity and Genotype Distribution among Samples Collected

**Figure 1:**
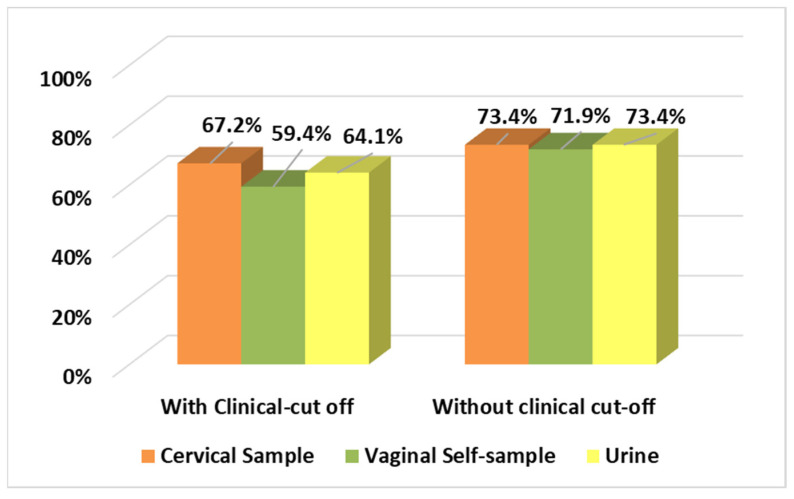
HPV positivity analysed considering and not considering clinical cut-off.

**Figure 2:**
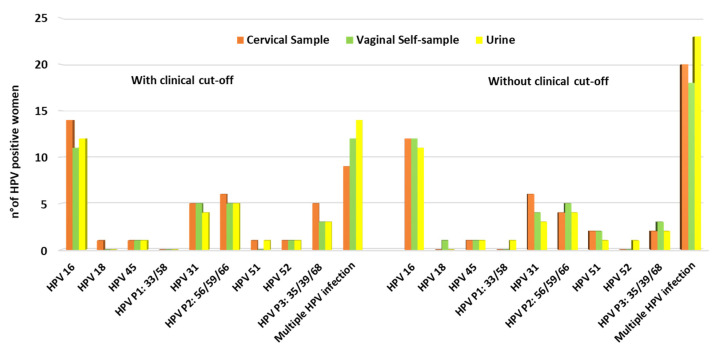
HPV genotype distribution analysed considering and not considering clinical cut-off.

HPV positivity results considering and not considering the clinical cut-off are reported in Figure 1. A very high rate of HPV positivity in cervical samples was found among the women enrolled: 67.2% (43/64) and 73.4% (47/64) considering and not considering the clinical cut-off, respectively. Considering just results obtained regarding HPV genotypes individually detected, HPV 16 was the most prevalent genotype identified among samples analysed, followed by HPV 31. Using the clinical cut-off, multiple genotype infections were observed in 14% (9/64), 18.7% (12/64), and 21.8% (14/64) of cervical, vaginal, and urine samples, respectively. Not considering the clinical cut-off, the percentages obtained were 31.2% (20/64), 28.1% (18/64), and 35.9% (23/64) for cervical, vaginal, and urine samples, respectively (Figure 2).

### 3.3. HPV Overall Positive Agreement between Cervical and Vaginal Self-Samples

Using clinical cut-offs of 38.3 Ct for HPV 16 and 34.2 Ct for other HR genotypes, compared to the cervical samples, the self-collected vaginal sample PPA (positive percentage agreement) was 36/43 = 83.7%; the NPA (negative percentage agreement) was 19/21 = 90.5%. The OPA was 85.9% (55/64), and the Cohen’s kappa was 0.699 (good agreement).

However, differences in collection procedures, preanalytical procedures, and the nature of the investigated samples may influence HPV detection rates on self-collected samples as compared to cervical specimens. Therefore, data analysis was performed without the clinical cut-off (accepting positivity up to Ct 40). Without the clinical cut-off, the PPA between self-collected vaginal samples and cervical samples was 45/47 = 95.7%; the NPA was 16/17 = 94.1%. The OPA was 95.3% (61/64), and the Cohen’s kappa was 0.882 (almost perfect agreement).

Using the package insert cut-offs of 38.3 Ct for HPV 16 and 34.2 Ct for other HR genotypes for the cervical samples and a cut-off of 40 Ct for the vaginal samples, to account for the excessive dilution, the self-collected sample PPA was 43/43 = 100%; the NPA was 18/21 = 85.7%. The OPA was 95.3%, and the Cohen’s kappa was 0.890 (almost perfect agreement). All results are reported in Table 2.

### 3.4. HPV Overall Positive Agreement between Cervical and First-Void Urine Samples

Using the clinical cut-offs, the PPA between first-void urine sample and cervical samples was 39/43 = 90.7% and the NPA 19/21 = 90.5%. The OPA was 87.5%, and the Cohen’s kappa was 0.79 (good agreement).

Without any clinical cut-off, the PPA was 43/47 = 91.5%; the NPA was 13/17 = 76.5%. The OPA was 95.3%, and the Cohen’s kappa was 0.680 (good agreement).

Using the package insert cut-offs of 38.3 Ct for HPV 16 and 34.2 Ct for other HR genotypes for the cervical samples and a clinical cut-off of 40 Ct for the urine samples, the PPA of self-collected urine samples was 42/43 = 97.7%; the NPA was 16/21 = 76.2%. The OPA was 90.6% (58/64), and the Cohen’s kappa was 0.776 (good agreement). The results are reported in Table 2.

### 3.5. Correlation between HPV Positivity and Clinical Outcome

**Figure 3:**
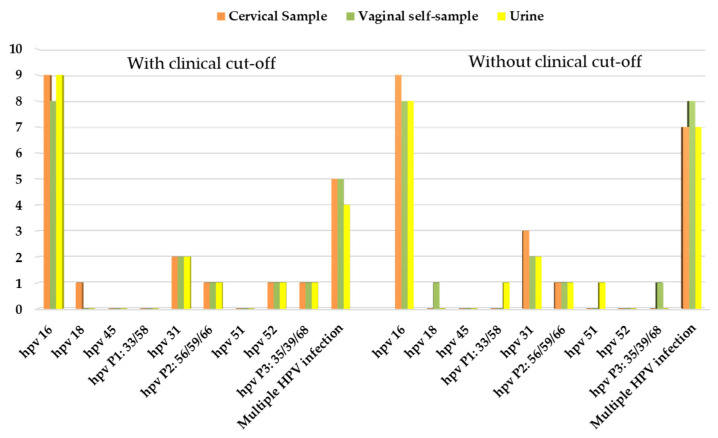
HPV genotypes’ distribution among women with abnormal colposcopy findings.

**Figure 4:**
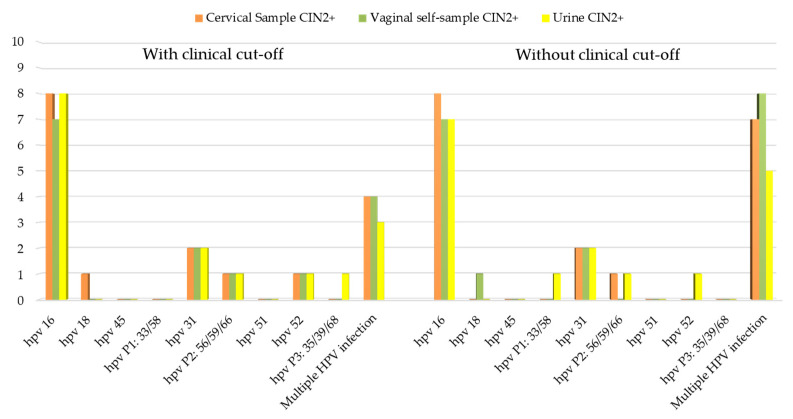
HPV genotypes’ distribution among women with CIN2+ lesions.

Comparing the results obtained from hrHPV testing with the clinical data, we evaluated the overall HPV positivity considering women with abnormal colposcopy findings and women with CIN2+ biopsy. Results for the cervical, vaginal, and urine samples with and without a clinical cut-off are presented in Table 3 and Table 4. In Figure 3 and Figure 4 are reported the HPV genotypes’ distribution among women with abnormal colposcopy (Figure 3) and among women with CIN2+ lesions (Figure 4).

## 4. Discussion

There is presently great interest in the use of self-collected samples as an alternative strategy in cervical cancer screening. The introduction of self-sampling in screening has been demonstrated to be more acceptable to women, resulting in improved participation in prevention programs [4,23].

Nowadays, several devices for self-sampling are commercially available, with different performances in sample collection [24,25,26]. The majority of HPV molecular assays are validated on cervical samples, the gold standard in cervical cancer screening [27]. However HPV assays may give different results when tested on self-taken vaginal and urine samples. Consequently, in order to obtain reproducible results, a specific HPV assay-sample-type validation is necessary [28].

The aim of this study was to evaluate accuracy of the BD Onclarity™ HPV assay on self-collected vaginal samples using a FLOQSwab^®^ and first-void urine using the Colli-Pee^®^ 20 mL device.

The results of the study showed a good overall positive agreement between self-collected specimens and clinician-collected samples, confirming data already reported in the expanded meta-analysis by Arbyn et al. [6,7]. Recently, a new meta-analysis of test agreement between HPV tests using self-taken vs. clinician-collected samples based on 26 studies (10,071 participants) was published, updating a previous meta-analysis on test accuracy for cervical precancers [29].

The validity of urine samples has been widely debated. On the one hand, this type of sample is easy to collect, overcomes some cultural barriers, and could be useful for surveillance in young populations [30]; on the other hand, the DNA in first-void urine is not always appropriately preserved, thus not allowing the obtaining of an adequate quantity of nucleic acids for HPV analysis [31]. Moreover, urine may collect cells from other nearby anatomical sites, accounting for the higher HPV detection rates and/or multiple infections when compared to cervical samples.

In this study, results from urine samples collected using the Colli-Pee^®^ 20 mL are comparable to results for cervical and vaginal samples, confirming—as previously reported in the literature—the validity of this device for the collection and storage of first-void urine using a preservative medium [15,16,17,18,32].

A limitation of the study is that the results come from samples collected from a population of women referred to colposcopy and not from a primary screening setting. However, the purpose of the study was to understand whether the BD Onclarity™ HPV assay could be used in combination with self-samples; hence, it was necessary to have a sufficient number of HPV-positive women to answer this question. For the same reason, previous studies aiming to evaluate HPV testing on clinician-collected vs. self-collected samples have been conducted in a colposcopy setting [18,33,34]. The other main limitation of this study is the small sample size, with participating women being enrolled at only one colposcopy centre. This analysis should therefore be considered a pilot study, and the data obtained could represent the starting point for further larger validation studies on new self-collection devices paired with the BD Onclarity™ HPV assay. Previous published reports also showed similar limitations regarding sample size as well as a lack of standardized preanalytical methods [14,20,32].

In the present study, vaginal swabs resuspended in 5.5 mL of PreservCyt and 20 mL of first-void urine samples were processed using the same preanalytical protocol as cervical samples by placing 0.5 mL of each sample’s starting volume in tubes containing 1.7 mL of BD preserve fluid. Differences in collection procedures, preanalytical procedures, and the nature of the investigated samples may influence HPV detection rates on self-collected samples as compared to cervical specimens, on which the BD Onclarity™ HPV assay protocol and clinical cut-offs had been previously validated. For this reason, we have analysed the data obtained both with the cut-offs set by the manufacturer for cervical samples and without the pre-set cut-offs, still obtaining comparable results in terms of concordance and agreement. However, the different results obtained underline the importance of considering using different testing protocols and analytical cut-offs based on the sample type.

No invalid samples were observed in this pilot study after retesting, irrespective of the sample type. The good sample adequacy observed for vaginal and first-void urine samples may have resulted from collection at the point of care and to reduced time from collection to laboratory testing.

HPV genotyping in self-collected samples represents another strength of the BD Onclarity™ HPV assay, as genotyping could be a good strategy for the triage of HR-HPV-positive women in order to identify those at greater risk of cervical cancer progression, such as HPV 16 and/or 18 positive women. The use of HPV genotyping assays could be very important, particularly for screening programs based on self-sampling due to the impossibility to performing cytology triage on the same sample. Moreover, HPV genotyping on self-taken samples could be helpful to look for persistence of the same HPV genotype at follow-up and as test-of-cure, without the need for a clinician-collected sample at each visit. The possibility of performing a reliable test to verify the success of surgical treatment or the risk of relapse on a self-taken sample can represent another great advantage for women to consider.

Due to the low number of positive samples, we did not perform a statistical analysis of specimens’ agreement considering specific HPV genotypes. However, these preliminary results showed a good concordance, especially for HPV 16, even if a further study enrolling a larger number of patients is necessary to obtain statistically significant results.

Up to now, the importance of self-sampling has been stressed to increase adherence to cervical cancer screening programs for women not participating due to socio-cultural or physical barriers. The SARS-CoV-2 pandemic has added another valuable reason to enhance the use of self-sample tests collected at home by women, thus avoiding the need to go to hospital and the risk of COVID exposure and infection [35].

## 5. Conclusions

Overall, data analysis without adjustment resulted in substantial agreement between the self-collected and clinic-collected samples, with PPA values of 83.7% and 90.7% for vaginal and urine samples, respectively. With correction for excess dilution in the sample preparation, there was almost perfect agreement, with PPA values of 100% and 97.7% for vaginal and urine samples, respectively. In conclusion, data from this pilot study are promising for the employment of the BD Onclarity™ HPV assay on vaginal samples and first-void urine samples, with an accuracy almost equivalent to clinician-collected cervical samples.

## Figures and Tables

**Table 1:** Clinical data of patients enrolled. Low-grade intraepithelial lesion (LSIL); atypical squamous cells of undetermined significance (ASCUS); high-grade intraepithelial lesion (HSIL); atypical glandular cells of undetermined significance (AGCUS); cervical intraepithelial neoplasia (CIN).

Pap Test Result	*n* (64)	%
ASCUS	15	23.4%
LSIL	24	37.5%
AGCUS	5	7.8%
ASCH	6	9.4%
HSIL	14	21.9%
Colposcopy Result	*n* (64)	%
ABNORMAL	23	35.9%
NORMAL	41	64.1%
Biopsy Result	*n* (23)	%
NEG	2	8.7%
CIN 1	3	13.0%
CIN 2	3	13.0%
CIN 3	14	60.9%
Cervical Cancer	1	4.3%

**Table 2:** Agreement in HPV detection of cervical and self-collected samples using different cut-off values.

	Vaginal Self-Sample				Urine			
Ct Cut-Off Value	PPA% (*n*)	PNA% (*n*)	OPA% (*n*)	κ	PPA% (*n*)	PNA% (*n*)	OPA% (*n*)	κ
<38.3 Ct for HPV 16 (for all sample types)	83.7% (36/43)	90.5% (19/21)	85.9% (55/64)	0.699	90.7% (39/43)	90.5% (19/21)	90.6% (58/64)	0.792
<34.2 Ct for the other HPVs (for all sample types)								
<40 Ct for HPV 16 and other HPVs (for all sample types)	95.7% (45/47)	94.1% (16/17)	95.3% (61/64)	0.882	91.5% (43/47)	76.5% (13/17)	87.5% (56/64)	0.680
<38.3 Ct for HPV 16 (for cervical samples)	100% (43/43)	85.7% (18/21)	95.3% (61/64)	0.890	97.7% (42/43)	76.2% (16/21)	90.6 (58/64)	0.776
<34.2 Ct for the other HPVs (for cervical samples)								
<40 Ct for HPV 16 and other HPVs (for self-samples)								

**Table 3:** HPV-positive women related to colposcopy results.

		With Clinical Cut-Off		Without Clinical Cut-Off	
	Colposcopy Results (Tot. 64)	Total HPV-Positive Women (*n*)	Total HPV-Positive Women (%)	Total HPV-Positive Women (*n*)	Total HPV-Positive Women (%)
Cervical Sample	ABNORMAL	20	31.3%	20	31.3%
	NORMAL	23	35.9%	25	39.1%
Vaginal self-sample	ABNORMAL	18	28.1%	21	32.8%
	NORMAL	20	31.3%	25	39.1%
Urine	ABNORMAL	18	28.1%	20	31.3%
	NORMAL	23	35.9%	27	42.2%

**Table 4:** HPV-positive women related to biopsy results.

		With Clinical Cut-Off		Without Clinical Cut-Off	
	Biopsy Results (Tot. 23)	Total HPV-Positive Women (*n*)	Total HPV-Positive Women (%)	Total HPV-Positive Women (*n*)	Total HPV-Positive Women (%)
Cervical Sample	CIN2+	17	73.9%	18	78.3%
	CIN2−	3	13.0%	4	17.4%
Vaginal self-sample	CIN2+	15	65.2%	18	78.3%
	CIN2−	3	13.0%	3	13.0%
Urine	CIN2+	16	69.6%	17	73.9%
	CIN2−	2	8.7%	3	13.0%
